# Case Report: Beating the assumed prognosis: homozygous familial hypercholesterolemia with unexpected long survival

**DOI:** 10.3389/fcvm.2025.1643771

**Published:** 2025-10-20

**Authors:** Lukáš Zlatohlávek, Jana Becherová Beňová, Tereza Foglarová, Tereza Dudková, Jaroslav A. Hubáček

**Affiliations:** ^1^ 3rd Department of Internal Medicine, Department of Endocrinology and Metabolism, 1st Faculty of Medicine, General University Hospital, Charles University, Prague, Czech Republic; ^2^ Experimental Medicine Centre, Institute for Clinical and Experimental Medicine, Prague, Czech Republic

**Keywords:** familial hypercholesterolemia, homozygous, phenotypic variability, treatment, longevity

## Abstract

**Background:**

Familial hypercholesterolemia (FH) is a common autosomal codominant genetic disorder, with heterozygous FH (HeFH) affecting approximately 1 in 310 individuals. FH is characterized by elevated low-density lipoprotein cholesterol (LDL-C) levels, which are typically twice those of unaffected individuals, and by a markedly increased risk of premature atherosclerotic cardiovascular disease (ASCVD). Homozygous FH (HoFH) is rarer and presents substantial phenotypic variability, with total cholesterol levels ranging from 13 to 55 mmol/L.

**Case presentations:**

We report three atypical cases of HoFH, with one patient being a homozygote for the c.1775G > A (p.Gly592Glu) variant and two patients being compound heterozygotes (c.340T > A/c.1775G > A, p.Phe114Ile/p.Gly592Glu and c.761A > C/c.910G > A, p.Gln254Pro/p.Asp304Tyr). All the patients presented with relatively mild clinical phenotypes, delayed diagnoses, and no evidence of early-onset ASCVD.

**Conclusions:**

These cases underscore the clinical heterogeneity of HoFH and challenge the prevailing assumption that HoFH uniformly results in severe cardiovascular outcomes. Personalized treatment strategies are essential for improving prognoses and quality of life of affected individuals.

## Introduction

Familial hypercholesterolemia (FH; OMIM ac. No. 143890) is a common autosomal codominant genetic disorder that is characterized by elevated low-density lipoprotein cholesterol (LDL-C) levels and an increased risk of premature atherosclerotic cardiovascular disease (ASCVD). It was first reported in 1982 (1), and recent meta-analyses estimate the prevalence of heterozygous FH (HeFH) to be approximately 1 in 310 individuals, with a 20-fold higher prevalence among those with premature ASCVD (2, 3). FH affects all ethnic groups globally.

Phenotypic expression is highly variable in patients with homozygous FH (HoFH), with total plasma cholesterol concentrations ranging from 13 to 55 mmol/L, whereas the concentration is approximately 10 mmol/L in patients with HeFH. Most cases are caused by pathogenic variants in the LDL receptor gene (*LDLR*; OMIM ac. No. 606945), although rare mutations in *APOB* (OMIM ac. No. 107730) or *PCSK9* (OMIM ac. No. 607786) have also been identified. The mutation spectrum is often population specific. Rare variants in other genes, such as *LIPA* (lysosomal acid lipase), *LIPG* (endothelial lipase), *LIPC* (hepatic lipase), and *PNPLA5* (patatin-like phospholipase domain-containing protein 5), can also result in FH-like phenotypes, usually in isolated pedigrees only (4). The additional genetic contributors to FH have yet to be fully elucidated.

Despite the significantly elevated HoFH-associated ASCVD risk (5), some patients exhibit prolonged survival, possibly because of early diagnosis, intensive lipid-lowering therapy, or unidentified protective genetic and environmental modifiers (6, 7).

## Methods and results

We present three clinically distinct cases of HoFH to highlight the phenotypic heterogeneity among these individuals. Patient characteristics and current pharmacological treatments are summarized in Table 1. Pathogenic variants in the *LDLR* gene were confirmed through targeted next-generation sequencing, which revealed two compound heterozygotes and one true homozygote. The common Czech *APOB* variant (p.Arg3527Gln) (8, 9) was excluded in all the cases. Importantly, in all three cases, HoFH was diagnosed incidentally during examinations for unrelated health issues. HoFH was primarily diagnosed by using clinical Duth Lipid Clinic Network criteria for Familial Hypercholesterolemia (10). Subsequently, genetic testing was introduced.

**Table 1:** General characteristics and treatment of examined subjects.

Parameter	Case 1	Case 2	Case 3
Sex	Male	Female	Female
Age	59	49	50
Age at HoFH diagnosis	41	15	23
Age at first DL treatment	41	15	30
Mutations			
DNA	c.340T > A/c.1775G > A	c.761A > C/c.910G > A	c.1775G > A
Protein	p.Ph114Ile/p.Gly592Glu	p.Asp304Asn/p.Gln254Pro	p.Gly592Glu
Smoking	Past, intensive	Never	Never
BMI	22,77	24,16	23,74
Diabetes	−	T2DM	−
Hypertension	+	−	−
SBP	124	143	135
DBP	84	78	71
Recent treatment	Piramil 5 MG	Stadamet 1000 mg	Rosuvastin 40 mg
	Godasal 100/50 MG	Loradur Mite 2,5/25 mg	Ezetimibe 10 mg
	Concor 5 mg	Zenon 40/10 mg	
	Pantoprazole 40 mg	Repatha 140 mg	
	Zenon 40 MG/10 MG		
	Praluent 150 mg		
*APOE* genotype	E3/E3	E2/E3	E3/E3

**Figure 1:**
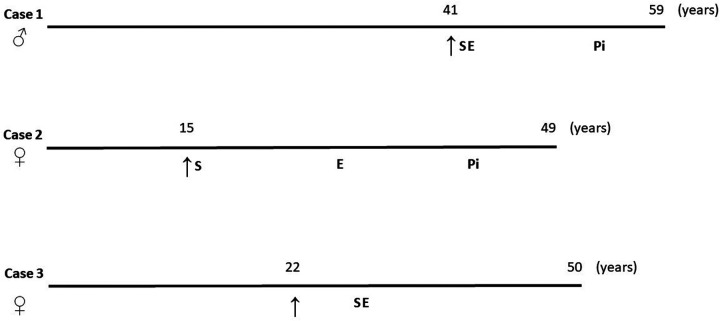
Diagnostic and treatment points of the HoFH subjects. ↑, time of the diagnosis; S, statin treatment was implemented; E, ezetimibe treatment was implemented; Pi, PCSK9 inhibitor treatment was implemented.

In cases one and two, the patients were gradually titrated to the maximal tolerated statin dose immediately after diagnosis. All the patients were administered ezetimibe as soon as it was released on the Czech market (Figure 1). Finally, the patients were among the first to be treated with PCSK9 inhibitors in the Czech Republic. In case three, the initiation of therapy was delayed. This delay reflected both patient-related factors — most notably, the patient's concerns about potential adverse effects — and broader challenges that could contribute to delayed treatment of dyslipidaemia, such as the asymptomatic nature of the disease, variability in clinical practice, and evolving access to newer therapies. Once these barriers were addressed, the patient was successfully started on lipid-lowering therapy with statins and ezetimibe.

Importantly, all the patients were employed and received full social support and assistance from other family members during the treatment. We did not observe any major limitations or deterioration in quality of life in our patients, associated with the disease.

General characteristics of cases are summarised in Table 1 and initial and last plasma lipid parameters and glycaemia in Table 2.

**Table 2:** Biochemical parameters of examined subjects without treatment and after medication.

Parameter	Case 1		Case 2		Case 3	
	Untreated	Treated	Untreated	Treated	Untreated	Treated
Total cholesterol (mmol/L)	16.8	6.6	16.7	4.7	10.4	4.1
LDL-C (mmol/L)	14.6	4.4	14.9	2.70	8.3	2.4
HDL-C (mmol/L)	1.94	1.44	1.5	1.50	1.83	1.55
TG (mmol/L)	2.79	1.76	0.56	1.14	0.62	0.69
Apo B (mmol/L)	3.90	1.5	3.37	0.87	2.53	1.07
Apo A1 (mmol/L)	1.27	1.25	n.a.	1.66	n.a.	1.43
Glycaemia (mmol/L)	5.4	5.2	5.4	5.4	4.10	4.5
Lp(a) (mg/dl)	n.a.	3.8	n.a.	0.05	n.a.	0.84

n.a., not analysed.

## Case presentations

### Case 1

A 59-year-old male who lived with his entire family, worked as a manual labourer, was a smoker, underwent a revascularization intervention, and had multiple comorbidities was diagnosed with HoFH at age 41 and presented with prominent xanthelasma palpebrarum. He was started on maximally tolerated lipid-lowering therapy, including a statin, ezetimibe, and later also PCSK9 inhibitor, along with antihypertensive treatment, which raised concerns about polypharmacy. At age 57, he underwent coronary artery bypass grafting because of significant stenoses in the ramus interventricularis anterior (60% and 50%), rami marginales sinisteri (70% and 50%), and arteria coronaria dextra (two lesions with 60% stenosis). Duplex ultrasonography revealed carotid plaques causing approximately 40% luminal narrowing.

**Figure 2:**
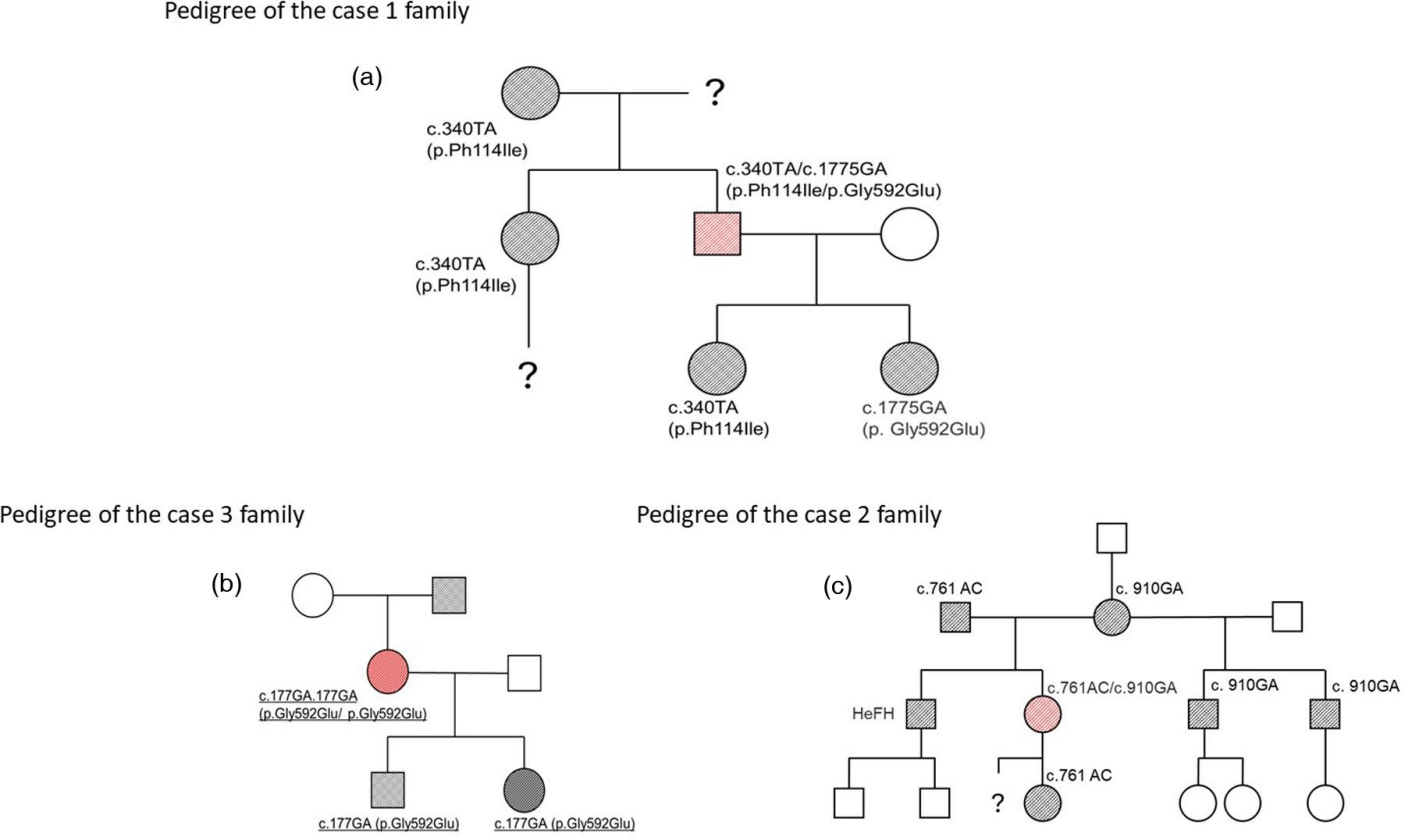
Family pedigrees of the examined FH homozygotes. squares: male; circles: female; empty symbol: unaffected individual; black-filled symbol: heterozygous FH; red-filled symbol: homozygous FH.

His medical history included surgical resection of a benign brain tumour at age 16 and peptic ulcer disease diagnosed at age 18, with resolution confirmed by normal gastroscopy in 2011. Genetic analysis revealed compound *LDLR* heterozygosity (c.340T > A [p.Phe114Ile] and c.1775G > A [p.Gly592Glu]). Family cascade screening revealed that his mother, sister, and one daughter were carriers of the c.340T > A variant, whereas a second daughter carried the c.1775G > A variant (Figure 2a).

### Case 2

A 49-year-old female sales assistant who lived in a fully functional family had type 2 diabetes mellitus (managed with oral hypoglycaemic agents) and arterial hypertension. She was diagnosed with HoFH at age 15, following hospitalization for surgical removal of Achilles tendon xanthomas. Lipid-lowering therapy was initiated promptly and currently includes a statin, ezetimibe, and a PCSK9 inhibitor, resulting in sustained total cholesterol levels below 5 mmol/L.

Genetic testing confirmed compound heterozygosity for the *LDLR* variants c.761A > C (p.Gln254Pro) and c.910G > A (p.Asp304Tyr). Both parents had clinically diagnosed and treated HeFH. Her brother had a clinical diagnosis of FH without genetic confirmation. Her daughter (carrier of c.761A > C) and two half-brothers (carriers of c.910G > A) were genetically confirmed HeFH heterozygotes (Figure 2b). No further genetic testing was performed on the other family members.

### Case 3

A 50-year-old female who was a graduated doctor of pharmacy was homozygous for the p.Gly592Glu *LDLR* variant, which is the most prevalent FH-associated mutation in the Czech population. She displayed a lipid profile characteristic of HeFH rather than HoFH. Lipid-lowering therapy with statins and ezetimibe was initiated more than seven years after diagnosis.

Her father, who had a history of hypercholesterolemia and asthma, underwent coronary artery bypass surgery at age 40 and died at 73. Both of her children were confirmed to be heterozygous carriers of the p.Gly592Glu variant (Figure 2c).

## Discussion

The incidental diagnosis of HoFH in our patients, with HoFH discovered during examinations for unrelated health conditions, contrasts with the typical diagnostic pathway involving early lipid profile screening, followed by genetic confirmation. These cases highlight the importance of clinical vigilance in detecting atypical presentations of HoFH. Two patients carried the p.Gly592Glu variant in the *LDLR* gene, which is the most common FH-causing mutation in the Czech population and accounts for approximately 20% of FH cases (8). In the patient who was homozygous for this variant, untreated lipid levels were more consistent with those of HeFH, and less intensive lipid-lowering therapy was sufficient to reduce the total cholesterol concentration to less than 5 mmol/L. These findings highlight the marked phenotypic variability among HoFH patients and align with those of previous reports describing a wide range of clinical outcomes.

As observed in our study, the presence of genetic mutations was not reflected in clear clinical results in all the patients. Case 3 is a clear example of this. The patient was diagnosed with HoFH at age 22 and was not treated during the subsequent seven years. Nevertheless, she is not suffering from CAD at age 50. In Patient 1, cigarette smoking certainly contributed to the accelerated atherosclerotic changes (surgically treated with a coronary bypass). Patient 2 had tendon xanthomas, which clearly indicated high cholesterol levels. Despite having type 2 diabetes mellitus, she has no manifestation of cardiovascular disease.

Thus, all our patients have a good chance of long-term survival, which is in agreement with observations of Marco-Benedí et al. (10), who reported that the mean age of death of HeFH patients was only four years less than that of the general population — a difference that was not statistically significant. Similarly, a long-term French cohort study of 53 HoFH patients reported only eight deaths over a 38-year follow-up period, despite that many individuals had been diagnosed before statins were available (7). Historical studies even suggested that FH may have conferred survival advantages in the preantibiotic era, possibly because of protection against infections (11). Elevated cholesterol levels have been associated with improved outcomes in patients with conditions such as sepsis, tuberculosis, and HIV (12–15). Nonetheless, in the context of modern lifestyles and therapeutics, FH mutations represent a significant cardiovascular risk.

Early FH diagnosis and the initiation of maximally tolerated lipid-lowering therapy undoubtedly remain critical, as untreated HoFH can lead to mortality as early as age four (6). Available treatments, including statins, ezetimibe, PCSK9 inhibitors, and LDL apheresis, can substantially reduce cholesterol levels (4). However, polypharmacy (which can lead to undesirable drug–drug interactions, treatment discontinuation and treatment failure) and more aggressive invasive interventions (which are also very time consuming) may negatively affect patients' quality of life, educational opportunities, and occupational outcomes (16).

Unfortunately, prenatal genetic screening for HoFH remains underutilized (17), despite the 25% risk of homozygosity when both parents are HeFH carriers.

A patient-tailored, holistic approach that balances clinical efficacy with overall well-being is therefore essential.

The long-term survival of our patients is also supported by the fact that in the Czech Republic, all inhabitants benefit from receiving the best possible treatment, regardless of their socioeconomic status. This is guaranteed through compulsory health insurance and generally very low patient contributions towards pharmacological treatment.

The limitation of our study is the relative small number of cases as well as the heterogeneity of these cases. As the diagnoses were made over 20 years ago, it is difficult to identify the environmental and/or genetic factors that contribute to long survival in subjects with HoFH. Whole genome sequencing of a large number of subjects could help to identify such protective mutations. Raising awareness of this disease is also extremely important, as in all three of our cases, the diagnosis was made by chance.

## Conclusion

The case studies presented herein help resolve outstanding gaps in the literature regarding HoFH. Despite the clear genetic criteria, the patients exhibited different clinical phenotypes, which were distorted by factors such as smoking in Patient 1. Genetic diagnosis enables the early identification of at-risk family members of probands, allowing us to maximally reduce their cardiovascular risk. In light of the steadily declining prices and increasing availability of genome-wide sequencing, the question arises as to whether genetic testing should be the primary diagnostic criterion for HoFH.

Although it is a monogenic disorder, homozygous familial hypercholesterolemia is characterized by substantial variability in lipid profiles and the onset and severity of atherosclerotic disease. Optimizing clinical outcomes while preserving the quality of life of patients requires a personalized treatment approach that considers environmental, metabolic, and possibly additional genetic factors beyond *LDLR* mutations.

## Data Availability

The raw data supporting the conclusions of this article will be made available by the authors, without undue reservation.
